# Protein misfolding diseases

**DOI:** 10.4155/fso.15.38

**Published:** 2015-09-01

**Authors:** Salvador Ventura

**Affiliations:** 1 Institute of Biotechnology & Biomedicine, Department of Biochemistry & Molecular Biology, Parc de Recerca UAB, Mòdul B, Universitat Autònoma de Barcelona, Barcelona, Spain

**Keywords:** amyloid, cancer, neurodegenerative disorders, protein aggregation, protein folding

Protein misfolding diseases include highly debilitating degenerative disorders like Alzheimer's and Parkinson's diseases [1]. The healthcare and financial burden linked to these pathologies has been steadily increasing over the past decade [2]. Actually, there is currently no efficient treatment for misfolding diseases as well as no reliable early diagnostic techniques for them [3]. It is known that, in many cases, the cytotoxic effect of misfolded proteins is exerted through their self-assembly into amyloid-like protein aggregates [4]. Unfortunately, the mechanisms responsible for the toxicity of protein aggregates are still not completely understood. The number of protein targets whose misfolding and aggregation is being shown to be associated with the onset of pathologic conditions is constantly increasing [5]. Since the development of degenerative disorders is expected to increase at a similar rate than life expectancy, it is likely that in the years to come misfolding diseases would become more common and prevalent than previously thought. We should be prepared to deal with such a dramatic scenario and join research efforts to understand the molecular mechanism that underlies these devastating disorders without further delay.

In this issue, we highlight recent advances in the study of protein misfolding disorders and their protein targets, but it is also described how different organisms exploit the amyloid fold for the functional and dynamic assembly of biological structures, in a review by S Fabio Falsone [6]. The complexity of misfolding diseases makes indispensable the use of different model organisms to model these pathologies; Kevin A Morano and coworkers describe how these models have been used for pharmacological and molecular modeling or cognitive assessment [7]. In all these models, as well as in humans, protein misfolding is antagonized by the heat shock response and the unfolded protein response, which are the major stress response pathways within protein quality control. Martin L Duennwald illustrates how cooperation between these different stress response pathways might play an important role in defining protein toxicity in Huntington's and other brain disorders [8]. In fact, the devastating CNS glioblastomas exploits the unfolded protein response to allow their continued uncontrolled growth, becoming thus a potential target for treatment intervention, as reviewed by Michael Graner [9]. Spinal and bulbar muscular atrophy, is a neuromuscular degenerative disease that, as in the case of the better characterized Huntignton's disease, arises from expansion of the polyglutamine repeats, in this case in the androgen receptor, indicating that similar sequential changes might lead to different disorders depending on the protein target in which they occur, as reported by Folake A Orafidiya and Iain J McEwan [10]. In fact, the propensity of a protein to form amyloid assemblies is imprinted in its sequence and can be read using computational approaches. An analysis of the aggregation propensity of neuroreceptors by Salvador Ventura and coworkers highlights the overlap between aggregation-prone regions and receptors interfaces and/or ligand-binding sites suggesting that anomalous interactions between them and protein aggregates might cause wrong signaling in brain neurones [11]. Cystic fibrosis is an inherited, misfolding disease impacting respiratory function. This disorder illustrates how the knowledge about the genetic and protein causes for misfolding diseases might lead to effective therapies that improve patient's life expectancy, as detailed in the review by Deborah O’Neil and Douglas Fraser-Pitt [12]. The increasing impact that misfolding diseases might have in our society is exemplified in the last article of this issue by Xavier Fernandez-Busquets and coworkers, where they discuss evidences supporting the role of amyloid assemblies in the pathobiology of malaria. The amyloid structures of the plasmodium parasite may become attractive targets to fight against this devastating disease [13]. This issue therefore brings together updated information on the causes underlying misfolding diseases, outlines the different pathways that might be taken in the next future for therapeutic intervention and provide fresh and provoking ideas on the role of amyloids in health and disease.
